# REscan: inferring repeat expansions and structural variation in paired-end short read sequencing data

**DOI:** 10.1093/bioinformatics/btaa753

**Published:** 2020-08-26

**Authors:** Russell Lewis McLaughlin

**Affiliations:** Smurfit Institute of Genetics, Trinity College Dublin, Dublin D02 PN40, Ireland

## Abstract

**Motivation:**

Repeat expansions are an important class of genetic variation in neurological diseases. However, the identification of novel repeat expansions using conventional sequencing methods is a challenge due to their typical lengths relative to short sequence reads and difficulty in producing accurate and unique alignments for repetitive sequence. However, this latter property can be harnessed in paired-end sequencing data to infer the possible locations of repeat expansions and other structural variation.

**Results:**

This article presents REscan, a command-line utility that infers repeat expansion loci from paired-end short read sequencing data by reporting the proportion of reads orientated towards a locus that do not have an adequately mapped mate. A high REscan statistic relative to a population of data suggests a repeat expansion locus for experimental follow-up. This approach is validated using genome sequence data for 259 cases of amyotrophic lateral sclerosis, of which 24 are positive for a large repeat expansion in *C9orf72*, showing that REscan statistics readily discriminate repeat expansion carriers from non-carriers.

**Availabilityand implementation:**

C source code at https://github.com/rlmcl/rescan (GNU General Public Licence v3).

## 1 Introduction

**Fig. 1.**
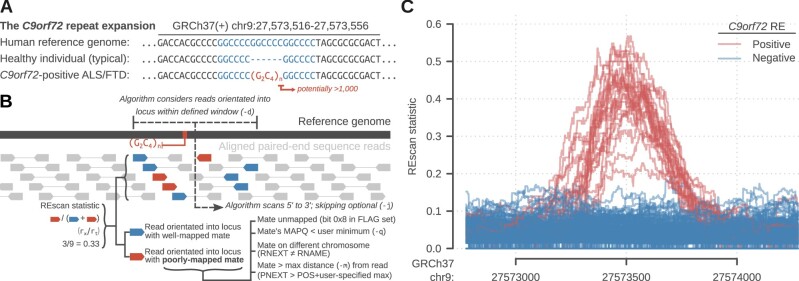
Repeat expansions and their inference using REscan. (**A**) The sequence and structure of the *C9orf72* repeat expansion (RE) in amyotrophic lateral sclerosis (ALS). (**B**) Principles of the REscan algorithm and its use on a paired-end SAM-format stream. (**C**) REscan statistics for 259 ALS cases in a 1.5 kb region surrounding the *C9orf72* RE

Repeat expansions (REs) are genetic variants characterized by an increase in the number of units in a tandem repeat sequence (Fig. 1A). REs are causative in many human diseases, especially nervous system disorders (van Blitterswijk *et al.*, 2012), suggesting convergent pathological mechanisms driven by long stretches of repetitive DNA in certain transcripts. The discovery of novel REs is thus important for understanding the biology of disease but can be challenging using conventional methods. For example, expanded alleles may not amplify by polymerase chain reaction (PCR) due to their length and, as often seen in neurological disorders, high GC content. With short read sequencing, expanded alleles longer than the read length lead to poor alignment and false-negative variant calls. However, with paired-end data, poorly mapped reads from the edges of expanded alleles often have mates with unique sequences that adequately align to the locus (‘anchoring’ reads). This is harnessed by algorithms such as ExpansionHunter (Dolzhenko *et al.*, 2017) and STRetch (Dashnow *et al.*, 2018); however, ExpansionHunter requires prior knowledge of the nature of the locus and the RE (which is not necessarily available for novel REs), and STRetch relies on computationally intensive realignment of reads to a reference genome supplemented with RE-containing decoy chromosomes. For pipelines aimed at efficient identification of novel REs from genome sequence data, a tool that reports observational data on unmapped reads without the need for *a priori* knowledge or preprocessing is useful. These data can then be used to statistically infer RE loci for technical and experimental validation. This article presents REscan, a command-line tool for exploratory analysis of paired-end sequence data to infer the locations of long REs and other structural variation.

## 2 Materials and methods

### 2.1 The REscan algorithm

REscan reads a position-sorted SAM-format stream from standard input and reports a straightforward, locus-wise statistic $r_{x}/r_{t}$, where *r_t_* is the total number of nearby reads orientated towards the locus and *r_x_* is the number with a poorly mapped mate (Fig. 1B). The window within which a mate is considered nearby is modifiable (default 200 bases), and a mate is considered poorly mapped if any of the following conditions are met:

- The next segment in template is unmapped (bit 0x8 in FLAG is set);
- The mate’s mapping quality is lower than 20 or a user-specified value;
- The mate is mapped to a different chromosome (RNEXT ≠ RNAME);
- The mate is more than 50 kb (user-definable) from the current read.

The latter two conditions allow for expanded alleles that are sufficiently homologous to a distal locus to be considered adequately mapped (empirical observations of RE alignments show this to frequently be true).

### 2.2 Testing with *C9orf72*-positive ALS cases

REscan was tested in whole-genome sequence data [Illumina HiSeq X 150 bp paired-end; aligned to GRCh37 using Isaac (Raczy *et al.*, 2013); mean coverage 36.9×] for 259 cases of amyotrophic lateral sclerosis (ALS) generated as part of Project MinE (van Rheenen *et al.*, 2018). All individuals had previously been screened for a large intronic hexanucleotide RE in *C9orf72* (forward strand sequence: GGCCCC) using repeat-primed PCR (Byrne *et al.*, 2012). Twenty-four individuals unambiguously exhibited 30 or more repeats and were classified positive for the RE.

## 3 Results and discussion

REscan is a lightweight command-line tool written in C with no special dependencies for installation. It is designed to be incorporated into a pipeline operating on a SAM-format stream (e.g. samtools view in.bam | rescan [options]) on whole genomes or targeted regions and outputs REscan statistics as a plain-text stream in variant call format. Output data can then easily be manipulated to serve downstream applications, e.g. merging and indexing for fast random access. Computational burden and memory requirements are low.

Figure 1C shows REscan output for 1.5 kb surrounding the *C9orf72* RE locus. Samples previously identified as positive for the RE yielded statistics that significantly exceeded those from negative individuals (mean ± s.d.: *C9orf72*+, 0.40 ± 0.085; *C9orf72-*, 0.035 ± 0.029; $P=2.0\times 10^{-15}$, Mann–Whitney–Wilcoxon test). For heterozygotes of a long *C9orf72* RE, the expected REscan statistic is 0.5; Figure 1C shows some variance for *C9orf72-*positive individuals, possibly reflecting variance in absolute RE length. These samples had been previously genotyped using repeat-primed PCR (Byrne *et al.*, 2012), which only yields reliable inference of up to 30–40 repeat units, rendering exact determination of the source of variance of REscan statistics difficult. However, this could be investigated using Southern blot or long-read sequencing with fresh high molecular weight DNA extracts.

### 3.1 Discovery pipeline for novel repeat expansions

REscan provides a flexible method for generating exploratory data to enable a discovery pipeline for novel REs, incorporating the following steps: (i) REscan is run individually per-sample across, for example, all transcribed regions; (ii) resulting data are appropriately processed (e.g. normalized/standardized) and statistically analysed in a regression framework or similar to identify candidate RE loci associated with the trait of interest; (iii) candidate loci are cross-referenced against structural variants called by alternative algorithms to filter false positives; (iv) the likely repeat motif is obtained from reference genome data, and common off-target alignment regions are identified from sequence data; (v) using candidates retained in step (iii) along with information obtained from step (iv), RE lengths are estimated in a more comprehensive framework such as ExpansionHunter; (vi) expanded alleles are validated experimentally using Southern blot, repeat-primed PCR or targeted long-read sequencing.
